# Improving Risk Stratification of Early Oral Tongue Cancer with TNM-Immune (TNM-I) Staging System

**DOI:** 10.3390/cancers13133235

**Published:** 2021-06-29

**Authors:** Alhadi Almangush, Ibrahim O. Bello, Ilkka Heikkinen, Jaana Hagström, Caj Haglund, Luiz Paulo Kowalski, Ricardo D. Coletta, Antti A. Mäkitie, Tuula Salo, Ilmo Leivo

**Affiliations:** 1 Department of Pathology, University of Helsinki, 00014 Helsinki, Finland; ibello@ksu.edu.sa (I.O.B.); ilkka.heikkinen@helsinki.fi (I.H.); jaana.hagstrom@hus.fi (J.H.); tuula.salo@helsinki.fi (T.S.); 2 Research Program in Systems Oncology, Faculty of Medicine, University of Helsinki, 00014 Helsinki, Finland; antti.makitie@helsinki.fi; 3 Institute of Biomedicine, Pathology, University of Turku, 20520 Turku, Finland; ilmo.leivo@utu.fi; 4 Faculty of Dentistry, Misurata University, Misurata 2478, Libya; 5 Department of Oral Medicine and Diagnostic Sciences, King Saud University College of Dentistry, Riyadh 11545, Saudi Arabia; 6 Department of Oral and Maxillofacial Diseases, University of Helsinki, 00014 Helsinki, Finland; 7 Research Programs Unit, Translational Cancer Medicine, University of Helsinki, 00014 Helsinki, Finland; caj.haglund@hus.fi; 8 Department of Oral Pathology and Radiology, University of Turku and Turku University Hospital, 20520 Turku, Finland; 9 Department of Surgery, University of Helsinki and Helsinki University Hospital, 00014 Helsinki, Finland; 10 Department of Head and Neck Surgery and Otorhinolaryngology, A.C. Camargo Cancer Center, São Paulo 01509-900, Brazil; lp_kowalski@uol.com.br; 11 Department of Oral Diagnosis, School of Dentistry, University of Campinas, Piracicaba, São Paulo 13083-970, Brazil; coletta@unicamp.br; 12 Department of Otorhinolaryngology—Head and Neck Surgery, University of Helsinki and Helsinki University Hospital, 00130 Helsinki, Finland; 13 Division of Ear, Nose and Throat Diseases, Department of Clinical Sciences, Intervention and Technology, Karolinska Institutet and Karolinska University Hospital, 17177 Stockholm, Sweden; 14 Cancer and Translational Medicine Research Unit, Medical Research Center Oulu, University of Oulu and Oulu University Hospital, 90220 Oulu, Finland; 15 Turku University Hospital, 20521 Turku, Finland

**Keywords:** oral tongue squamous cell carcinoma (OTSCC), tumor-infiltrating lymphocytes (TILs), TNM AJCC 8, TNM-Immune staging, survival

## Abstract

**Simple Summary:**

Some patients with early-stage oral tongue cancer suffer from poor survival. The currently used classification requires further improvement to better predict the prognosis. Immune-related parameters (such as assessment of infiltrating lymphocytes) can be used as a modifier for the classification and that can aid in improving the prognostication. We included 290 cases of early-stage oral tongue cancer in this study. Lymphocytes were scored and divided as low or high and incorporated in the traditional tumor-node-metastasis (TNM) classification to form our proposed TNM-Immune staging system. The TNM-Immune staging system allowed for a significant distinction between T1 and T2. The TNM-Immune staging system showed a powerful ability to identify cases with poor survival. TNM-Immune staging forms a step towards a more personalized classification of early-stage oral tongue cancer.

**Abstract:**

Although patients with early-stage oral tongue squamous cell carcinoma (OTSCC) show better survival than those with advanced disease, there is still a number of early-stage cases who will suffer from recurrence, cancer-related mortality and worse overall survival. Incorporation of an immune descriptive factor in the staging system can aid in improving risk assessment of early OTSCC. A total of 290 cases of early-stage OTSCC re-classified according to the American Joint Committee on Cancer (AJCC 8) staging were included in this study. Scores of tumor-infiltrating lymphocytes (TILs) were divided as low or high and incorporated in TNM AJCC 8 to form our proposed TNM-Immune system. Using AJCC 8, there were no significant differences in survival between T1 and T2 tumors (*p >* 0.05). Our proposed TNM-Immune staging system allowed for significant discrimination in risk between tumors of T1N0M0-Immune vs. T2N0M0-Immune. The latter associated with a worse overall survival with hazard ratio (HR) of 2.87 (95% CI 1.92–4.28; *p* < 0.001); HR of 2.41 (95% CI 1.26–4.60; *p =* 0.008) for disease-specific survival; and HR of 1.97 (95% CI 1.13–3.43; *p =* 0.017) for disease-free survival. The TNM-Immune staging system showed a powerful ability to identify cases with worse survival. The immune response is an important player which can be assessed by evaluating TILs, and it can be implemented in the staging criteria of early OTSCC. TNM-Immune staging forms a step towards a more personalized classification of early OTSCC.

## 1. Introduction

Oral tongue squamous cell carcinoma (OTSCC) is the most common and aggressive SSC of the oral cavity. The most clinically relevant parameter in the classification of OTSCC is the tumor-node-metastasis (TNM) staging system which evaluates tumor size (T), lymph node status (N) and distant metastasis (M) [1]. For early OTSCC, however, the clinical behavior of many cases is unpredictable based on the current TNM classification only. Even with the improved performance of the eighth edition of the American Joint Committee on Cancer (AJCC 8) staging system of oral cancer [2], many of the early stage (i.e., T1-2N0M0) cases show an aggressive behavior that associates with cancer-related mortality. Therefore, multimodality treatment is necessary for such cases. However, a decision to apply aggressive treatments is a clinical dilemma as early OTSCCs are usually treated with a single modality approach. Thus, there is a need for further refinement of the staging system for the early OTSCC.

Data accumulating on the significance of adaptive immune response, regardless of histologic grade, were reported for different cancers and this approach has shown promising results that can be useful in routine practice [3,4]. However, in early OTSCC, the clinical utility of immune response is not yet established. Of note, tumor immunology research has shown that tumors at the same stage may present with extreme differences in their pre-existing adaptive immunity [5]. Remarkably, good reproducibility in the assessment of tumor-infiltrating lymphocytes (TILs) in hematoxylin and eosin (HE) stained sections was recently confirmed in different tumors [6,7,8]. Furthermore, the density of TILs was reported to influence tumor progression and patient survival in a significant way in many early-stage cancers, including early OTSCC [7,9,10,11,12]. Thus, the incorporation of TILs in the staging system as a marker of immune response might improve risk stratification in early-stage OTSCC and aid in the identification of high-risk cases in this population. The aim of this multicenter study is to introduce a proposal of a TNM-Immune (TNM-I) staging system for early-stage OTSCC.

## 2. Materials and Methods

This study was conducted with the permission of the National Supervisory Authority for Welfare and Health in Finland and the Brazilian Human Research Ethics Committee. A total of 290 cases treated for OTSCC at the five Finnish university hospitals or at the A-C Camargo Cancer Center in São Paulo, Brazil, and re-staged according to the criteria of the TNM AJCC 8 were included in this study. We included only naïve tumors that were treated by surgical resection. Our data did not include cases with immunosuppression or previous chemotherapy. As our analysis considered only early-staged OTSCC, all cases were either T1N0M0 or T2N0M0 according to the criteria of the TNM AJCC 8. Our analysis aimed to compare the prognostic value of the classic TNM AJCC 8 with that of our proposed TNM-Immune staging system in predicting three survival endpoints: overall survival (defined as the time from surgery to death of any cause), disease-specific survival (defined as the time from surgery to death due to OTSCC), and disease-free survival (defined as the time from surgery to recurrence).

Stromal TILs were defined as stromal areas occupied by lymphocytes as explained in our previous study [7]. The assessment was in line with recent standardized recommendations for the evaluation of TILs in HE-stained sections [13]. In brief, each slide was scanned with a low magnification of ×5 to ×10 objectives and then the average of TILs was estimated at a higher magnification of ×20 to ×40 objectives. From our previous research [7], the score of stromal TILs at the invasive front was clinically the most relevant and therefore was considered in our analysis. To avoid focusing on hot spots, the average of TILs, semi-quantitatively assessed in % (e.g., 5%, 10%, 20%, 30%) was considered. Two authors (IOB and IH) conducted the assessments independently, and a good inter-observer agreement was observed (Kappa value = 0.75).

Our proposed TNM-Immune classification is designed based on the TNM AJCC 8 and includes the status of preexisting immunity as revealed by evaluation of TILs as follows:

T1: Tumor ≤ 2 cm, ≤5 mm depth of invasion, and TILs > 20%.

T2: Tumor ≤ 2 cm, DOI > 5 mm and ≤10 mm; or tumor > 2 cm but ≤4 cm, and ≤10 mm DOI. TILs should be ≤20% (i.e., TILs infiltration should not exceed 20% of the stromal area), otherwise downstaging is necessary.

Statistical analysis: We used SPSS Statistics software (version 25) for all statistical analyses. Pearson Chi-Square test (two-sided) was used to analyze the correlation between the TNM-Immune staging system and the traditional parameters. The Kaplan–Meier method and log-rank test were used to create survival curves. The univariable and multivariable analyses were conducted using a cox proportional hazard regression. A *p* value of less than 0.05 was considered significant. Age, gender, perineural invasion, tumor grade, and TNM stage were included in the multivariable analysis in addition to our proposed TNM-Immune staging system.

## 3. Results

The demographic and clinicopathologic data of 290 patients are shown in Table 1. The mean age of patients was 62 years. The median follow-up time was 57 months. There were 152 (52.4%) men and 138 (47.6%) women. According to the classic TNM AJCC 8 staging system, 88 (30.3%) cases were of stage T1, and 202 (69.7%) were of T2 that were included in this study. The same cohort was re-classified according to our proposed TNM-Immune staging system and then there were 243 (83.8%) of stage T1N0M0-Immune and 47 (16.2%) were T2N0M0-Immune. At the end of the follow-up, 145 (50%) cases were alive, 78 (26.9%) cases had developed recurrence, 55 (19%) had died of OTSCC, and 90 (31%) died of other causes.

The cross-tabulation (Table 1) showed a significant relationship between a higher TNM-Immune stage and aggressive histologic tumor characteristics including an infiltrative pattern of invasion (*p =* 0.037) and perineural invasion (*p =* 0.013). However, there was no significant association between TNM-Immune stage and tumor grade, patient age or gender (*p >* 0.05). In univariate analysis of TNM AJCC 8 (Table 2), a hazard ratio (HR) of 1.15 (95% CI 0.79–1.68; *p =* 0.473) was reported for overall survival, a HR of 1.36 (95% CI 0.73–2.53; *p =* 0.339) was reported for disease-specific survival, and a HR of 0.69 (95% CI 0.43–1.09; *p =* 0.108) for disease-free survival. The multivariable analyses summarized in Table 2 showed no prognostic difference (*p >* 0.05) between cases that were early-stage OTSCC as classified by AJCC 8.

**Figure 1:**
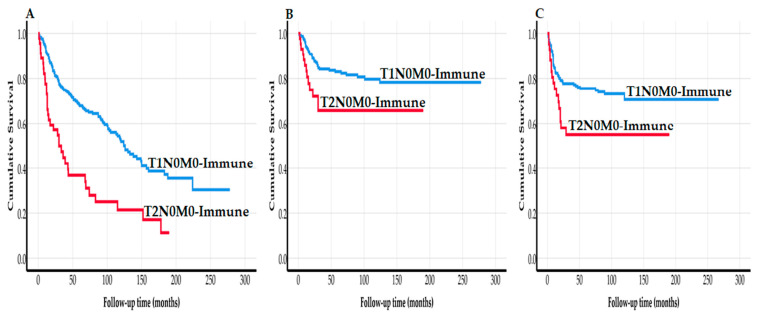
Correlation between TNM-Immune staging system and overall survival ((**A**): *p* < 0.001), disease-specific survival ((**B**): *p* = 0.010), and disease-free survival ((**C**): *p* = 0.012) in 290 patients treated for early oral tongue cancer.

In the analysis of the same cohort (*n* = 290), the newly proposed TNM-Immune staging system allowed for a significant risk stratification between cases staged as T1N0M0-Immune compared to cases of T2N0M0-Immune. We found that T2N0M0-Immune associated significantly with a worse outcome with a HR of 2.52 (95% CI 1.71–3.71; *p* < 0.001) for overall survival, a HR of 2.22 (95% CI 1.19–4.15; *p =* 0.012) for disease-specific survival, and a HR of 1.97 (1.13–3.43; *p =* 0.017) for disease-free survival. The worse prognosis of T2N0M0-Immune cases was confirmed in multivariable analyses with a HR of 2.87 (95% CI 1.92–4.28; *p* < 0.001) for overall survival; HR of 2.41 (95% CI 1.26–4.60; *p =* 0.008) for disease-specific survival; and HR of 1.97 (95% CI 1.13–3.43; *p =* 0.017) for disease-free survival. Furthermore, Kaplan–Meier survival curves (Figure 1A–C) showed a statistically significant difference in survival between cases of T1N0M0-Immune vs. T2N0M0-Immune. In a further analysis, T2N0M0-Immune cases associated with a high risk of local recurrence with a HR of 2.91 (95% CI 1.44–5.88; *p =* 0.003) in univariable analysis and a HR of 2.79 (1.35–5.76; *p =* 0.006) in the multivariable analysis. With regard to regional recurrence, however, there was no significant difference (*p >* 0.05) between T1N0M0-Immune and T2N0M0-Immune.

## 4. Discussion

The TNM classification is the main tool for prognostication and treatment decision-making in oral tongue cancer. For early-stage OTSCC, single-modality treatment is widely considered as the treatment of choice. However, some cases of early OTSCC were reported with a dramatically worse outcome including mortality due to such tumors. Even after improvement in the performance of AJCC 8, the challenge remains in deciding which early-stage OTSCCs need surgical resection only and which need multimodality treatment. In the present study, we introduced a powerful TNM-Immune (TNM-I) staging system that can be routinely implemented in the clinical setting to identify aggressive cases of early OTSCC.

Tumor cells interact with the surrounding immune microenvironment and this interaction is a major player during tumorigenesis and it has a valuable clinical significance [14]. Evasion of immune destruction is a hallmark of cancer progression [15]. The inclusion of a parameter in the staging system representing the immune status is of great importance in the development of personalized treatment approaches [5,10]. The incorporation of an immune parameter as part of the TNM tumor classification (i.e., TNM-Immune staging) was recently discussed with regard to different cancers [10,16,17]. However, which immune marker/parameter should be considered in TNM-Immune staging can vary from one tumor type and location to another based on accumulated evidence. As an example, in colorectal cancer, an immunoscore is based on the assessment of infiltration of two lymphocyte populations recognized by immunostains to CD3 and CD8, while in breast cancer overall assessment of TILs in HE-stained slides was suggested [17]. Furthermore, the location assessed may be different: in colorectal cancer, both stromal and intra-epithelial areas were important, while in breast cancer the stromal area was clinically the most relevant area [17].

TILs have been studied in different subsites of head and neck cancer [18]. The sub-population analysis of TILs requires specific immunostaining which is not routinely ordered in clinical practice of oral SCC. In addition, a recent meta-analysis found that CD4 and CD8 expression were not significant predictors in oral SCC [19]. Of note, an increasing body of evidence indicates that stromal TILs assessed in HE-stained sections can serve as a valuable marker to reveal the immune response in many tumor types including OTSCC [7,20,21]. In addition, automated analysis of TILs in HE-stained sections was reported recently with a promising prognostic value for oral SCC [22]. As the current classification of early-stage OTSCC (T1-T2N0M0) mainly depends on the T class, we proposed in this study to further implement the immune status of the tumor (as indicated by the assessment of TILs in the stromal compartment). Accordingly, T class will include tumor diameter, depth of invasion and the pre-existing immune response. In the present study, this proposal identified the cases associated with poor survival (Table 2) and could therefore benefit from more aggressive treatments. More importantly, those cases were not identified by the classic TNM AJCC 8 as shown in Table 2. The significance of TILs as an indicator of pre-existing adaptive immunity, and the possibility of assessing TILs in HE-stained sections, as well as the high reproducibility of TILs score, makes the TNM-Immune staging system readily applicable for daily use. It requires only a minimal effort of the pathologist and no additional costs.

A few limitations of this study should be mentioned. It is a retrospective study and examined the proposed TNM-Immune staging system in only one subsite of the oral cavity (i.e., the oral tongue). The relationship between TNM-Immune and some variables including neck dissection, surgical margins, or adjuvant treatments was not analyzed in this study due to lack of data in many cases. In addition, a digital scoring of the immune response was not considered. These limitations need to be addressed in future studies.

## 5. Conclusions

In early-stage OTSCC, there is a lack of evidence on the TNM-Immune classification. Our study reports for the first time the significance of including an immune parameter as a part of the staging system of early-stage OTSCC. TILs score, as a valuable indicator of the immune response, can routinely be included in the clinical practice and seems worth implementing in the staging system. Classification of OTSCC tumors staged as T1-T2N0M0 can be refined by TNM-Immune staging to recognize cases at a high risk of a worse outcome. It is necessary to initiate prospective studies, preferably multi-institutional, as a further step toward the introduction of TNM-Immune staging as part of routine prognostication and therapeutical decision-making in early OTSCC.

## Figures and Tables

**Table 1:** Relationship between our proposed TNM-Immune (TNM-I) system and the clinicopathologic characteristics of 290 patients treated for early-stage (AJCC 8) oral tongue cancer.

Parameter	Total (%)	T1N0M0-Immune	T2N0M0-Immune	*p* Value
		Number (%) 243 (83.8%)	Number (%) 47 (16.2%)	
Age				0.844
≤60	121 (41.7%)	102 (84.3%)	19 (15.7%)	
>60	169 (58.3%)	141 (83.4%)	28 (16.6%)	
Gender				0.164
Men	152 (52.4%)	123 (80.9%)	29 (19.1%)	
Women	138 (47.6%)	120 (87.0%)	18 (13.0%)	
WHO Grade				0.102
Well-differentiated	95 (32.8%)	83 (87.4%)	12 (12.6%)	
Moderately-differentiated	125 (43.1%)	107 (85.6%)	18 (14.4%)	
Poorly-differentiated	70 (24.1%)	53 (75.7%)	17 (24.3%)	
Perineural invasion				0.013
No	254 (87.6%)	218 (85.8%)	36 (14.2%)	
Yes	36 (12.4%)	25 (69.4%)	11 (30.6%)	
Pattern of invasion				0.037
Cohesive	72 (24.8%)	66 (91.7%)	6 (8.3%)	
Infiltrative	218 (75.2%)	177 (81.2%)	41 (18.8%)	
TNM AJCC 8				0.140
T1N0M0	88 (30.3%)	78 (88.6%)	10 (11.4%)	
T2N0M0	202 (69.7%)	165 (81.7%)	37 (18.3%)	

**Table 2:** Overall survival, disease-specific survival and disease-free survival analyses of 290 patients of early oral tongue cancer (AJCC 8). The survival analyses include the routinely evaluated classification (TNM AJCC 8 and WHO Grading) and our proposed TNM-immune classification.

Parameter	Overall Survival		Disease-Specific Survival		Disease-Free Survival	
	Univariable Analysis HR (95% CI) *p* Value	Multivariable Analysis HR (95% CI) *p* Value	Univariable Analysis HR (95% CI) *p* Value	Multivariable Analysis HR (95% CI) *p* Value	Univariable Analysis HR (95% CI) *p* Value	Multivariable Analysis HR (95% CI) *p* Value
Age	*p* < 0.001	*p* < 0.001	*p* = 0.012	*p* = 0.010	*p* = 0.015	*p* = 0.014
≤60	Reference	Reference	Reference	Reference	Reference	Reference
>60	2.32 (1.63–3.31)	2.49 (1.73–3.61)	2.12 (1.18–3.79)	2.19 (1.20–4.01)	1.81 (1.12–2.93)	1.86 (1.13–3.05)
Gender	*p* = 0.164	*p* = 0.022	*p* = 0.339	*p* = 0.433	*p* = 0.571	*p* = 0.888
Men	Reference	Reference	Reference	Reference	Reference	Reference
Women	0.79 (0.57–1.10)	0.67 (0.47–0.94)	1.29 (0.76–2.20)	1.25 (0.72–2.17)	1.14 (0.73–1.78)	0.97 (0.61–1.54)
WHO Grade	*p* = 0.233	*p* = 0.219	*p* = 0.316	*p* = 0.157	*p* = 0.798	*p* = 0.497
Well	Reference	Reference	Reference	Reference	Reference	Reference
Moderate	1.34 (0.92–1.96)	1.39 (0.96–2.05)	1.64 (0.87–3.12)	1.85 (0.97–3.53)	1.09 (0.65–1.86)	1.15 (0.68–1.96)
Poor	1.02 (0.65–1.61)	1.18 (0.74–1.88)	1.41 (0.66–2.99)	1.78 (0.82–3.85)	1.23 (0.68–2.22)	1.44 (0.79–2.64)
Perineural invasion	*p* = 0.086	*p* = 0.233	*p* = 0.478	*p* = 0.743	*p* = 0.224	*p* = 0.13
No	Reference	Reference	Reference	Reference	Reference	Reference
Yes	1.47 (0.95–2.28)	1.32 (0.84–2.09)	1.31 (0.62–2.78)	1.14 (0.53–2.46)	1.47 (0.79–2.71)	1.65 (0.87–3.16)
TNM AJCC 8	*p* = 0.473	*p* = 0.697	*p* = 0.339	*p* = 0.239	*p* = 0.108	*p* = 0.075
T1N0M0	Reference	Reference	Reference	Reference	Reference	Reference
T2N0M0	1.15 (0.79–1.68)	1.08 (0.73–1.61)	1.36 (0.73–2.53)	1.48 (0.77–2.83)	0.69 (0.43–1.09)	0.64 (0.39–1.05)
TNM-Immune	*p* < 0.001	*p* < 0.001	*p =* 0.012	*p* = 0.008	*p* = 0.015	*p* = 0.017
T1N0M0-Immune	Reference	Reference	Reference	Reference	Reference	Reference
T2N0M0-Immune	2.52 (1.71–3.71)	2.87 (1.92–4.28)	2.22 (1.19–4.15)	2.41 (1.26–4.60)	1.96 (1.14–3.37)	1.97 (1.13–3.43)

Abbreviations: HR: Hazard ratio; CI: Confidence interval.

## Data Availability

The datasets used in this study are available from the corresponding author upon a reasonable request.
